# The Eucalyptus Tonoplast Intrinsic Protein (TIP) Gene Subfamily: Genomic Organization, Structural Features, and Expression Profiles

**DOI:** 10.3389/fpls.2016.01810

**Published:** 2016-11-30

**Authors:** Marcela I. Rodrigues, Agnes A. S. Takeda, Juliana P. Bravo, Ivan G. Maia

**Affiliations:** ^1^Department of Genetics, Institute of Biosciences of Botucatu, São Paulo State UniversityBotucatu, Brazil; ^2^Department of Physics and Biophysics, Institute of Biosciences of Botucatu, São Paulo State UniversityBotucatu, Brazil; ^3^Institute of Biotechnology, São Paulo State UniversityBotucatu, Brazil

**Keywords:** aquaporin, tonoplast intrinsic protein, gene structure, gene expression, abiotic stress, Eucalyptus

## Abstract

Plant aquaporins are water channels implicated in various physiological processes, including growth, development and adaptation to stress. In this study, the Tonoplast Intrinsic Protein (TIP) gene subfamily of Eucalyptus, an economically important woody species, was investigated and characterized. A genome-wide survey of the *Eucalyptus grandis* genome revealed the presence of eleven putative TIP genes (referred as *EgTIP*), which were individually assigned by phylogeny to each of the classical TIP1–5 groups. Homology modeling confirmed the presence of the two highly conserved NPA (Asn-Pro-Ala) motifs in the identified EgTIPs. Residue variations in the corresponding selectivity filters, that might reflect differences in EgTIP substrate specificity, were observed. All *EgTIP* genes, except *EgTIP5.1*, were transcribed and the majority of them showed organ/tissue-enriched expression. Inspection of the *EgTIP* promoters revealed the presence of common *cis*-regulatory elements implicated in abiotic stress and hormone responses pointing to an involvement of the identified genes in abiotic stress responses. In line with these observations, additional gene expression profiling demonstrated increased expression under polyethylene glycol-imposed osmotic stress. Overall, the results obtained suggest that these novel *EgTIPs* might be functionally implicated in eucalyptus adaptation to stress.

## Introduction

Aquaporins are membrane channels implicated in the transfer of water and small solutes across cell membranes (reviewed in Maurel et al., 2015). In plants, aquaporins fall into at least seven different subfamilies that reflect their main subcellular localization (reviewed in Maurel et al., 2015). Members of the Tonoplast Intrinsic Protein (TIP) subfamily are generally targeted to the vacuolar membrane and known to facilitate water transport across this subcellular compartment. Parallel to their role as water channels, TIP isoforms can also translocate glycerol, hydrogen peroxide (H_2_O_2_) (reviewed in Maeshima, 2001) and urea (Liu et al., 2003), and enhance vacuolar membrane permeability to ammonia (Loqué et al., 2005; Kirscht et al., 2016). Specificity of substrate transport is mainly determined by four amino acid (aa) residues present at specific positions of the so-called aromatic/arginine (ar/R) selectivity filter, which acts as a size-exclusion barrier (Sui et al., 2001; Wu et al., 2009).

Reports on the expression of a set of TIP genes revealed differential regulation in response to environmental constraints, especially drought and salinity, and to abscisic acid (ABA) (Alexandersson et al., 2005; Boursiac et al., 2005; Rodrigues et al., 2013; Regon et al., 2014). Supporting the importance of these proteins in plant stress adaptation, TIPs were shown to improve drought and salt tolerance when overexpressed in transgenic plants (Peng et al., 2007; Khan et al., 2015; Li and Cai, 2015). Intriguingly, TIP overexpression also positively affected plant growth and biomass production (Lin et al., 2007; Peng et al., 2007; Sade et al., 2009; Khan et al., 2015; Li and Cai, 2015). Although some discrepancies exist, since overexpression phenotypes displaying sensitivity to abiotic stress have been observed (Wang et al., 2011), the current consensus is that TIPs are engaged in the regulation of important physiological processes that contributes to plant growth and adaptation to stress (reviewed in Afzal et al., 2016). In this context, a recent role for different TIP isoforms in facilitating lateral root emergence in *Arabidopsis thaliana* was demonstrated (Reinhardt et al., 2016).

A systematic genome-wide analysis conducted in 10 different plant species, including both monocts and dicots, identified at least 100 genes encoding TIPs (Regon et al., 2014). In *A. thaliana*, the *TIP* subfamily is composed of 10 members that are phylogenetically classified into five subgroups (Johanson et al., 2001). In forest and fruit tree species, genome-wide surveys revealed the presence of 17 *TIP* genes in *Populus trichocarpa* (Gupta and Sankararamakrishnan, 2009) and *Hevea brasiliensis* (Zou et al., 2015a), while 10 and 11 genes were identified in *Vitis vinifera* and *Citrus sinensis*, respectively (Shelden et al., 2009; Martins et al., 2015). Intriguingly, a preliminary study identified a relatively small number of *TIP* genes (five in total) in the Eucalyptus genome (Rodrigues et al., 2013). Taking into consideration the evolutionary history of the eucalyptus genome (Myburg et al., 2014), such a comparatively low number of genes is unexpected and requires additional investigation.

To gain further insights into the functional/structural attributes of the TIP subfamily of Eucalyptus, in the present study we undertook a genomic survey for additional members. This effort led to the identification of six novel genes (referred to as *EgTIP*), indicating that the *EgTIP* subfamily encompasses 11 members. In parallel, we investigated the phylogeny, structural features and expression patterns across different eucalyptus organs/tissues and in response to osmotic stress of the entire gene subfamily. The results obtained provide an interesting framework for future studies aiming to elucidate the roles of TIPs in woody species.

## Materials and Methods

### Plant Material

Freshly harvested vegetative and reproductive organs/tissues (1 month-old plantlets; pool of leaves of different ages; roots from 1 and 6 month-old plantlets; stems from 1 and 6 month-old plantlets; vascular cambium samples from 6 year-old trees; flower; flower buds; and fruits) were obtained from *Eucalyptus grandis* essentially as described previously (Rodrigues et al., 2013). After harvesting, fresh samples were frozen immediately in liquid nitrogen until total RNA extraction. Two-month-old plantlets of a commercial clone of *E. grandis*, kindly provided by Suzano Papel e Celulose SA, Brazil, were used in the osmotic stress assays.

### *In silico* Identification and Characterization of the Eucalyptus TIP Genes (*EgTIP*)

To identify members of the TIP gene subfamily in Eucalyptus, annotated TIP sequences from *Arabidopsis* (Johanson et al., 2001; Alexandersson et al., 2005) were used as queries in BLAST searches against the *E. grandis* BRASUZ1 genome assembly (v2.0) available at Phytozome^1^. To refine our analysis, additional BLAST searches were performed using previously identified poplar TIP sequences (Gupta and Sankararamakrishnan, 2009) as drivers. The identified gene products were subsequently validated using the Pfam domain annotation (Finn et al., 2016). The exon/intron organization of the mined *EgTIP* genes was determined using the Eucalyptus gene models annotated in Phytozome and the Gene Structure Display Server^2^ (GSDS v2.0). The existence of duplication of the *EgTIP* genes was investigated using the locus-search module of the Plant Genome Duplication Database^3^ (PGDD). The ratio of non-synonymous (Ka) to synonymous (Ks) substitution rates of evolution were also calculated using PGDD. Searches for the presence of putative *cis*-regulatory elements within the *EgTIP* promoter regions were carried out using the Plant Care and PLACE databases. Predictions of the subcellular location of the EgTIP proteins were performed using Plant-mPLoc^4^ (Chou and Shen, 2010).

### Phylogenetic Analyses

The deduced aa sequences of the identified EgTIPs were aligned with known TIPs from *A. thaliana*, *P. trichocarpa*, *Quercus petraea*, and *V. vinifera* using CLUSTALX2^5^. Phylogenetic relationships were inferred using the neighbor-joining method with 1000 bootstrap replicates implemented by the MEGA 7 software package^6^. Branches with <50% bootstrap support were collapsed. Accession numbers of the sequences used in the phylogenetic analyses are as follows: AtTIP1.1 (At2g36830), AtTIP1.2 (At3g26520), AtTIP1.3 (At4g01470), AtTIP2.1 (At3g16240), AtTIP2.2 (At4g17340), AtTIP2.3 (At5g47450), AtTIP3.1 (At1g73190), AtTIP3.2 (At1g17810), AtTIP4.1 (At2g25810), AtTIP5.1 (At3g47440), QqTIP1.1 (JQ846274), QqTIP2.1 (JQ846275), QqTIP2.2 (JQ846276), VvTIP1.1 (GSVIVP 00018548001), VvTIP1.2 (GSVIVP00000605001), VvTIP1.3 (GSVIVP00022146001), VvTIP1.4 (GSVIVP00024394001), VvTIP2.1 (GSVIVP00034350001), VvTIP2.2 (GSVIVP00012703001), VvTIP3.1 (GSVIVP00013854001), VvTIP4.1 (GSVIVP00032441001), VvTIP5.1 (GSVIVP00029946001), VvTIP5.2 (GSVIVP00019170001), PtTIP1.1 (549212), PtTIP1.2 (833283), PtTIP1.3 (822504), PtTIP1.4 (656044), PtTIP1.5 (667870), PtTIP1.6 (589502), PtTIP1.7 (558321), PtTIP1.8 (828458), PtTIP2.1 (548890), PtTIP2.2 (645978), PtTIP2.3 (817166), PtTIP2.4 (676397), PtTIP3.1 (584517), PtTIP3.2 (811826), PtTIP4.1 (561759), PtTIP5.1 (414059), PtTIP5.2 (423803). The Phytozome IDs of the mined *E. grandis* TIPs are: EgTIP1.1 (Eucgr.K02438), EgTIP1.2 (Eucgr.J00051), EgTIP1.3 (Eucgr.B02403), EgTIP1.4 (Eucgr.J02074) EgTIP2.1 (Eucgr.F03054), EgTIP2.2 (Eucgr.D02090), EgTIP2.3 (Eucgr.D02507), EgTIP3.1 (Eucgr.K02339), EgTIP3.2 (Eucgr.H00668), EgTIP4.1 (Eucgr.C02914), and EgTIP5.1 (Eucgr.K00164).

### Molecular Modeling

The deduced aa sequences of EgTIPs representing each TIP group (EgTIP1.1; EgTIP2.1; EgTIP3.1; EgTIP4.1, and EgTIP5.1) were employed for the construction of the models. These structures were predicted based on the alignment data generated by the program Phyre2 that uses homology detection methods to build 3D models (Kelley et al., 2015). Considering the quality of the structure, the crystallographic structure of the *A. thaliana* aquaporin TIP2.1 (AtTIP2.1) at 1.18 Å resolution (PDB ID: 5I32, Chain A) (Kirscht et al., 2016) was chosen as template. The program MODELLER 9v16 (Sali and Blundell, 1993; Marti-Renom et al., 2000) was used to generate the protein models and rank them according to the DOPE (Discrete Optimized Protein Energy) score. The best TIP models were selected based on the stereochemical parameters using the program RAMPAGE (Lovell et al., 2003). The ConSurf Server (Landau et al., 2005; Ashkenazy et al., 2010) was used for conservation analysis and all images were generated using the PyMOL program (Schrödinger, 2011).

### PEG-Imposed Osmotic Stress

The Eucalyptus plantlets (two months old) were transferred to hydroponic culture by means of a floating system and maintained in an aerated Hoagland’s nutrient solution (75%) at pH 6.0 (osmotic potential of -0.1 MPa) before stress imposition. After an acclimatization period, 50% of the tested plantlets were stressed by adding 215 g of polyethylene glycol 8000 (PEG; Sigma, USA) to 1 L of culture medium to induce osmotic stress, while the remaining were maintained in Hoagland’s nutrient solution (75%) throughout the assay as control. The osmotic potential of the PEG 8000-treated solution was -0.6 MPa (WP4-T, Decagon Devices, Inc., England). A compressed air system was used to homogenize the solutions in both the tray and the PEG container to avoid anoxia of the roots.

Both control and stress treatments comprised 15 plantlets per tray, and three trays per treatment (control/treated plantlets), representing three biological replications. To carry out the gene expression analyses, the roots, stems and leaves from three randomized plantlets per tray were collected after 6 h (short-term), 12 h (medium-term), and 24 h (long-term) of PEG-treatment. Organ samples from untreated control plantlets were simultaneously collected at each time-point. To reduce plant-to-plant variation, each collected group of organ samples was pooled before RNA extraction.

### *EgTIP* Expression Analyses

The relative expression of the *EgTIP* genes was assessed using quantitative real-time RT-PCR (RT-qPCR). Total RNA extraction and cDNA synthesis were performed as previously described (Rodrigues et al., 2013). The qPCR analyses were carried out using Power SYBR Green Master Mix (Applied Biosystems) and a StepOnePlus Real Time PCR System (Applied Biosystems). The cycling conditions were as follows: 5 min at 95°C, followed by 45 cycles of 15 s at 95°C and 60 s at 60°C. Each reaction was performed in triplicate in a total volume of 10 μl, and contained 20 ng of cDNA and 0.2 μM of each *EgTIP*-specific primer (**Supplementary Table S1**). Among five selected *E. grandis* reference genes (*Actin*, *GAPDH*, *Cdk8*, *Transcription elongation factor s-II*, and *Aspartyl-tRNA synthetase*; de Oliveira et al., 2012) tested, *actin* was the most stable and thus employed as endogenous control (**Supplementary Table S1**; Goicoechea et al., 2005). Cycle threshold (Ct) values were obtained for each sample, and relative quantification was determined using the 2^-ΔΔCt^ method as described (Livak and Schmittgen, 2001) Amplification efficiencies were derived from the amplification plots using the program LinRegPCR (Ramakers et al., 2003). A value of two was used in calculations. For the expression analysis under osmotic stress, the relative expression of each *EgTIP* at a given time point was determined as the fold change of its expression under treated condition relative to its expression under control condition.

Relative expression data were analyzed using the Relative expression software tool (REST 2009) and differences with *p*-values <0.05 were considered statistically significant. The heatmaps were created using the function heatmap.2 from plot package at R environment. For the organ/tissue-specific gene expression analyses, normalization was performed using 1-month-old plantlets as control sample as previously reported (Yu et al., 2014). According to Yu et al. (2014), these plantlets represent a highly stable and less variable sample that contain the main investigated organs/tissues. Additional expression analyses were performed using publicly available RNA-Seq data^7^ generated for six different vegetative organs/tissues (young and mature leaves, shoot tips, phloem, immature xylem and xylem) from a 6-year-old *E. grandis* X *E. urophylla* hybrid clone (Mizrachi et al., 2010). In this case, transcript abundances were expressed as units of normalized FPKM (fragments per kilobase of exon per million fragments mapped), that were used for heatmap construction.

## Results

### Identification and Analysis of the Eucalyptus TIP Genes

**FIGURE 1:**
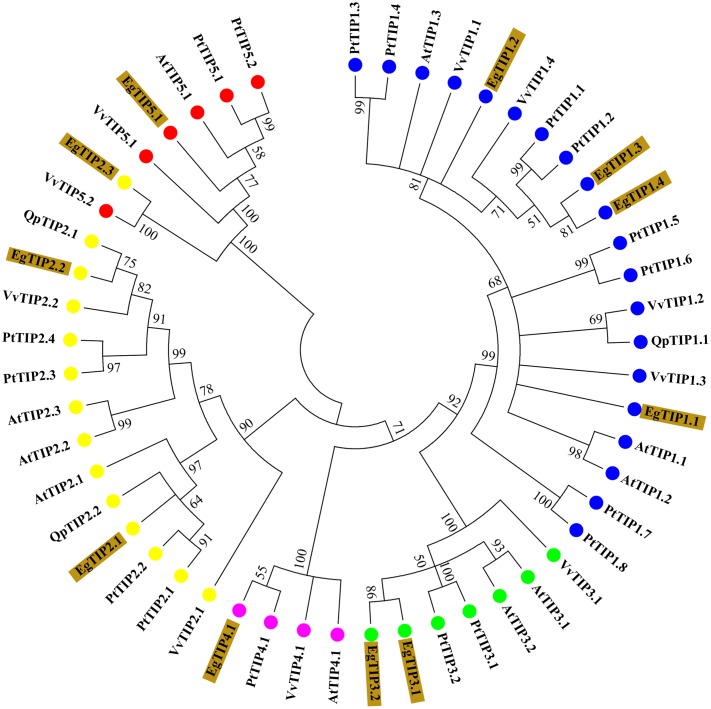
**Phylogenetic relationships between *Eucalyptus grandis* (Eg), *Arabidopsis thaliana* (At), *Populus trichocarpa* (Pt), *Vitis vinifera* (Vv), and *Quercus petreae* (Qp) TIP proteins.** The neighbor-joining unrooted phylogenetic tree was constructed using 1000 bootstrap replicas. Numbers next to each node indicate bootstrap values. Branches with <50% bootstrap support were collapsed. The identified EgTIPs are highlighted in brown while each TIP group (1-5) is indicated by colored circles.

BLAST searches in the *E. grandis* genome identified 11 putative TIP coding sequences (**Table 1**), thus indicating that the Eucalyptus TIP subfamily has 11 members (refereed as *EgTIP*) and not 5 as previously suggested (Rodrigues et al., 2013). To analyze in more detail, the evolutionary relationships between the identified EgTIPs and those present in other plant species, an unrooted phylogenetic tree was constructed using the predicted protein sequences (**Figure 1**). Based on their phylogenetic relationships, the EgTIPs could be individually assigned to each of the classical TIP1–5 groups (Johanson et al., 2001), and were named accordingly (EgTIP1.1, EgTIP1.2, EgTIP1.3 and EgTIP1.4; EgTIP2.1, EgTIP2.2 and EgTIP2.3; EgTIP3.1 and EgTIP3.2; EgTIP4.1; and EgTIP5.1). In general, the corresponding groups, with the exception of group 1, were supported by moderate to high bootstrap values. The identity at the aa level varied according to the TIP group considered: 79–88% identity between EgTIP1s, 76%–47% identity between EgTIP2s and 77% between EgTIP3s. Taking into account the tree topology, EgTIP2.3 was grouped with known TIP5 isoforms, being closely related to VvTIP5.2 (61% identity at the aa level). Nevertheless, the inspection of the ar/R selectivity residues of both VvTIP5.2 and EgTIP2.3 (see below) revealed a closer relationship with TIP2 isoforms. Therefore, based on its selectivity filter, EgTIP2.3 was assigned here as a TIP2 as originally proposed in Phytozome instead of a TIP5. In fact, the small subgroup formed by VvTIP5.2 and EgTIP2.3 is branched between the groups encompassing TIP5.1 and TIP2 isoforms. In this regard, EgTIP2.3 shares 44 and 47% aa sequence identity with EgTIP5.1 and EgTIP2.2, respectively. As observed in other plant species, the majority of the identified EgTIPs belongs to group 1 (four members) followed by group 2, 3, and 5 with two members each. The phylogenetic tree also revealed the presence of closely related gene pairs (*EgTIP1.3*/*EgTIP1.4* and *EgTIP3.1*/*EgTIP3.2*, for example), suggesting possible gene duplication events.

**Table 1:** Structural and subcellular localization analysis of the EgTIPs.

							Ar/R selectivity filter^∗^					NPA motif^∗∗^		Froger’s positions				
			
Protein	Ch^a^	Size	pI	MW (kDa)	TM^b^	Loc^c^	H2	LC	H5	LE1	LE2	LB	LE	P1	P2	P3	P4	P5
EgTIP1.1	3	260	5.56	26	6	Vacuole	H	F	I	A	V	NPA	NPA	T	S	A	Y	W
EgTIP1.2	10	252	4.68	26	6	Vacuole	H	F	I	A	V	NPA	NPA	T	S	A	Y	W
EgTIP1.3	2	252	4.79	26	6	Vacuole	H	F	I	A	V	NPA	NPA	T	S	A	Y	W
EgTIP1.4	10	251	5.16	25.8	6	Vacuole	H	F	I	A	V	NPA	NPA	T	S	A	Y	W
EgTIP2.1	6	248	5.52	25	6	Vacuole	H	H	I	G	R	NPA	NPA	T	S	A	Y	W
EgTIP2.2	4	250	5.12	25	6	Vacuole	H	H	I	G	R	NPA	NPA	T	S	A	Y	W
EgTIP2.3	4	259	6.78	26.4	6	Cell membrane/vacuole	H	G	F	G	R	NPA	NPA	A	S	A	H	
EgTIP3.1	11	262	6.7	27.5	6	Vacuole	H	F	I	A	L	NPA	NPA	T	A	A	Y	W
EgTIP3.2	8	259	6.04	27.8	6	Vacuole	S	L	I	A	R	NPA	NPA	T	A	A	Y	W
EgTIP4.1	3	243	5.91	25.3	6	Vacuole	H	H	I	A	R	NPA	NPA	T	S	A	Y	W
EgTIP5.1	11	256	6.81	25.7	6	Cell membrane/vacuole	N	F	V	G	C	NPA	NPA	T	S	A	Y	W


*^∗^Ar/R: aromatic/arginine; H2: transmembrane helix 2; LC: loop C; H5: transmembrane helix 5; LE: loop E; ^∗∗^NPA: Asn-Pro-Ala. ^a^Chromosome location of the corresponding gene. ^b^Number of transmembrane (TM) helices predicted by TMpred. ^c^Best possible subcellular localization predicted by Plant-mPloc.*

**FIGURE 2:**
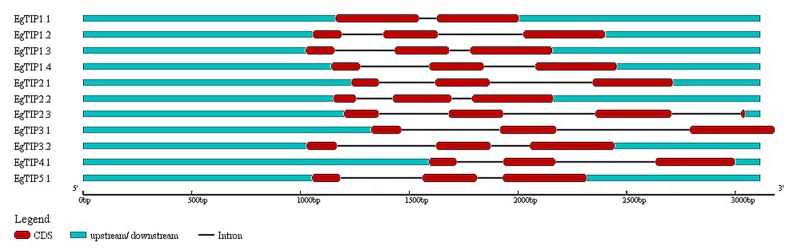
**Schematic representation of the exon/intron structures of the *EgTIP* genes.** The exons are represented by red rectangles and introns by thin black lines. The untranslated regions are depicted in blue. Lengths in nucleotides are shown at the bottom. The graphic representation was displayed using GSDS v2.0.

Analysis of the genomic structure of the annotated *EgTIP* genes revealed a two-intron/three-exon organization, except for *EgTIP1.1* that contained two exons disrupted by a single intron (**Figure 2**). These introns showed variations in length and position. Consistent with this, most members of the TIP subfamily of dicot species share such a well-conserved two-intron/three-exon pattern (Regon et al., 2014). Regarding their chromosomal distribution, the *EgTIP* genes are located on seven (2, 3, 4, 6, 8, 10, and 11) out of the eleven eucalyptus chromosomes (**Table 1**). In general, one or two genes are found per chromosome. In this context, *EgTIP1.1* and *EgTIP4.1* (on 3), *EgTIP1.2* and *EgTIP1.4* (on 10), *EgTIP2.2* and *EgTIP2.3* (on 4) and *EgTIP3.1* and *EgTIP5.1* (on 11) are among those located on the same chromosome.

An additional locus search at the PGDD website was performed to evaluate possible *EgTIP* gene duplication. According to the resulting data, the following gene pairs were observed: *EgTIP1.3*/*EgTIP1.4*, *EgTIP2.1*/*EgTIP4*, *EgTIP3.1*/*EgTIP3.2*, and *EgTIP2.2*/*EgTIP2.3*. Among them, only *EgTIP2.2* and *EgTIP2.3* are located on the same chromosome (4), and may possibly represent tandemly duplicated genes. To investigate the mechanisms involved in gene duplication evolution after divergence, we calculated the Ka/Ks ratio of each *EgTIP* gene pairs. The results (**Supplementary Table S2**) revealed that the Ka/Ks ratios of all four *EgTIP* gene pairs were less than 0.4, implying that these genes had undergone purifying selection pressure with limited functional divergence after duplication.

### Structural Features of the Identified EgTIPs

The deduced EgTIP proteins ranged in size from 243 (EgTIP4.1) to 262 (EgTIP3.1) aa residues, with molecular weights (MW) and theoretical isoelectric point (pI) values raging between 25 and 27.8 kDa and 4.68 and 6.81, respectively (**Table 1**). These proteins are all predicted to contain six transmembrane α-helices (H1–H6) and five loops. Concerning their sub-cellular localization, all EgTIPs are predicted to be located in the vacuole, with the exception of EgTIP5.1 and EgTIP2.3, that are also localized at the cell membrane (**Table 1**).

To evaluate structural similarities and differences, theoretical models for EgTIPs representing each classical TIP group were constructed using a comparative modeling approach and the crystallography structure of AtTIP2.1 at 1.18Å resolution (Kirscht et al., 2016) as a template. The sequences shared 53–85% aa identity and 90–95% of coverage. As expected, all models contained the six α-helices and interhelical loop regions as seen throughout the aquaporin family. The best models showed good stereochemical quality with 96.9% of the residues in favored regions, 2.6–1.7% and 0–0.9% (corresponding to two residues) in allowed and disallowed regions, respectively (**Supplementary Table S3**).

**FIGURE 3:**
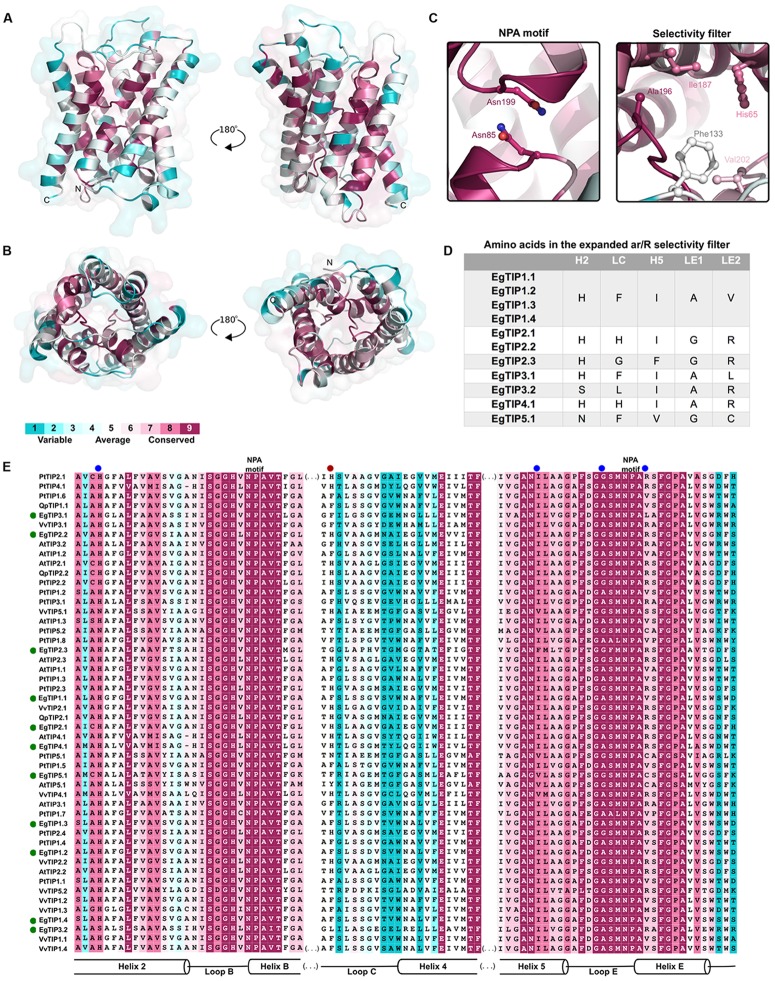
**Conservation of the EgTIP proteins.** The protein sequences employed for the construction of the phylogenetic tree were used and residues were colored according to conservation. **(A)** Lateral view of EgTIP1.1 shown in ribbon representation in two positions. **(B)** Top and bottom views of EgTIP1.1. **(C)** Residues of the NPA motif and selectivity filter shown in stick representation. **(D)** Residues of the expanded selectivity filter. **(E)** Multiple sequence alignment colored according to the surfacing mapping of the EgTIP1.1 model. The green and blue dots indicate EgTIP sequences and residues of the selectivity filter, respectively. The red dot corresponds to the residue from the expanded selectivity filter.

Residue conservation was reflected by surface mapping of the aa sequences of several plant TIPs over the generated EgTIP1.1 model (**Figure 3**). An overall evaluation of the EgTIP models detected the dual NPA (Asn-Pro-Ala) motifs conserved in all structures (**Figures 3A,C**; **Supplementary Figure S1**). In contrast, the key residues of the selectivity filter (positions H2, H5, LE1, and LE2) displayed some variations between the modeled proteins (**Table 1**; **Figures 3B,C**; **Supplementary Figure S1**). In all EgTIP1 and EgTIP2, the tetrad of the ar/R selectivity filter was composed of HIAV and HIGR, respectively (**Table 1**; **Supplementary Figure S1**). Despite showing phylogenetic relationships with TIP5 isoforms (**Figure 1**), the selectivity filter of EgTIP2.3 (HFGR) resembles the one found overall in group 2 members (HIGR; **Figure 3E**). Remarkably, EgTIP2.3 lacks the I residue in the H5 position, but has instead an F residue that preserves the hydrophobicity. Interestingly, the closest homologue of EgTIP2.3, VvTIP5.2, also harbors a classical TIP2-like selectivity filter (HIGR). For the other EgTIPs, slight residue variations at specific positions were observed (**Table 1**; **Figure 3D**). According to previous reports, a V residue is typically found at the LE2 position of TIPs from group 1, while an R is found in TIPs from groups 2, 3, and 4, whereas a C residue is present in all group 5 TIPs (Wallace and Roberts, 2004; Gupta and Sankararamakrishnan, 2009). In this regard, EgTIP3.1 harbors an atypical L residue in this position, while an R residue in EgTIP2.3 replaces the usual C residue (**Table 1**; **Figure 3D**). In addition, compared to the other EgTIPs, EgTIP3.2 and EgTIP5.1 harbor changes in the H2 position (S and N, respectively, instead of H), while EgTIP5.1 and EgTIP2.3 display variations in H5 (V and F, respectively, instead of I) (**Table 1**; **Figure 3D**).

Further comparison of the EgTIP-deduced aa sequences with those of other plant TIPs revealed that TIP members of groups 1 and 2 have the most conserved selectivity filters, including the H residue located in loop C (LC) of the proposed extended ar/R filter (Kirscht et al., 2016) (**Figures 3D,E**). VvTIP1.3 from *V. vinifera*, that possesses an M residue instead of a V in the LE2 position, is the only exception. The members of group 3 have most conserved residues in the H5 and LE1 positions and small variations in the H2 and LE2 positions. On the other hand, group 4 members have differences mainly in the LC and H5 positions, while EgTIP5.1 have the most variable LC positions.

### The *EgTip* Genes Show Enriched Organ/Tissues Profiles

**FIGURE 4:**
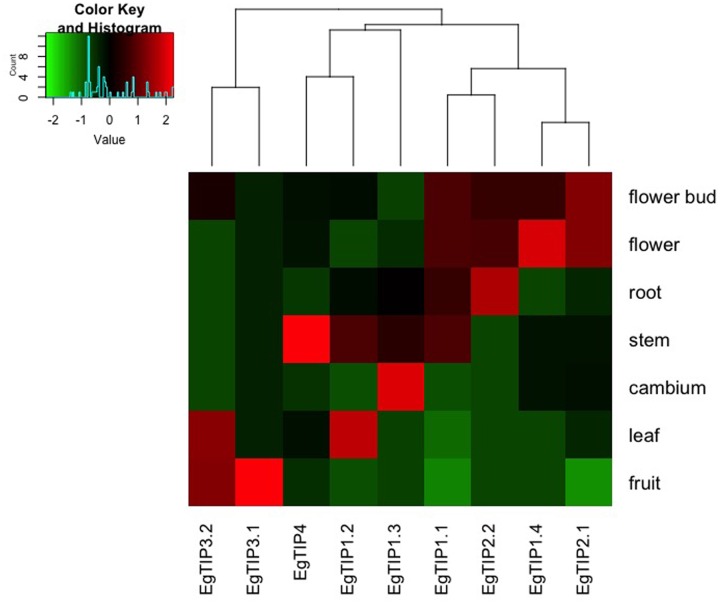
**Expression profiles of *EgTIP* genes in different *E. grandis* organs/tissues.** The heatmap was generated employing the relative expression of each *EgTIP* as determined by RT-qPCR normalized with the control sample (1-month-old plantlets). Red and green colors denote relatively high and lower expression compared to control, respectively. Genes were clustered according to their expression profiles. All samples were analyzed in triplicate.

To investigate the spatiotemporal expression profiles of the identified *EgTIP* genes, we first assessed their relative expression over a panel of different Eucalyptus organs/tissues using RT-qPCR. As shown in **Figure 4**, all members of the *EgTIP* subfamily, except *EgTIP5.1*, were transcribed. Among the genes investigated, solely *EgTIP1.1* had a relatively broad distribution among organs/tissues, being expressed at moderate levels in the roots, stems, flowers and flower buds. In contrast, the other *EgTIP* genes surveyed showed distinctive organ/tissue-enriched expression. Of the genes grouped in the so-called group 1, *EgTIP1.3* was preferentially expressed in vascular cambium and stems, *EgTIP1.4* in flowers and flower buds, and *EgTIP1.2* in leaves and stems. Of group 2, *EgTIP2.1*, and *EgTIP2.2* were both moderately expressed in flowers and flower buds, but recapitulating previous data (Rodrigues et al., 2013), *EgTIP2.2* was also highly expressed in roots. Of note, *EgTIP2.3* was barely detected in leaves and stems and was not included in the heatmap. The duplicated gene pair *EgTIP3.1* and *EgTIP3.2* shared a similar fruit-enriched expression, but *EgTIP3.2* was also detected at moderate levels in leaves. Finally, *EgTIP4.1* was prominent in the stems.

**FIGURE 5:**
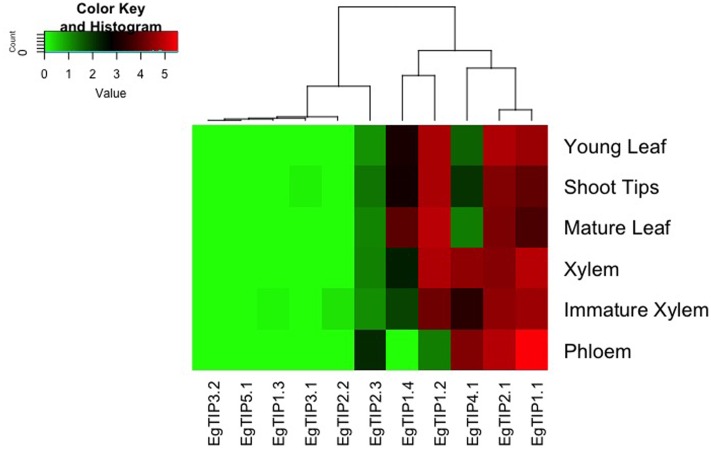
***EgTIP* expression profiles determined using the RNA-Seq data from different *E. grandis* organs/tissues.** The heatmap was generated employing normalized FPKM values available at Phytozome. The color scale is the same as in **Figure 4**.

*EgTIP* spatial expression patterns were further examined employing publicly available RNA-Seq data (FPKM values) from six different organs/tissues of a hybrid clone of Eucalyptus (Mizrachi et al., 2010). Interestingly, this dataset included vegetative and vascular tissue samples (such as phloem, xylem, and shoot tips) that were not represented in the previously performed RT-qPCR assays. According to the digital expression profiles generated (**Figure 5**), a wide expression pattern was observed for *EgTIP2.1* and *EgTIP1.1*, which were detected at moderate to high levels in all organs/tissues examined. A similar pattern was observed for *EgTIP1.2*, except for its very low expression level in the phloem. According to our RT-qPCR assays, this gene was predominantly expressed in leaves (**Figure 4**). On the other hand, *EgTIP1.4* was detected at moderate levels in mature leaves and at lower levels in young leaves and in shoot tips, while *EgTIP4.1* was prominent in vascular tissues (phloem and xylem). This is consistent with the preferential expression of *EgTIP4.1* in the stems as indicated by RT-qPCR (**Figure 4**). Contrasting with *EgTIP5.1* that was not expressed, *EgTIP2.3* was observed at very low levels in the phloem. The other *EgTIP* genes, including those mainly expressed in reproductive organs as determined by RT-qPCR, were almost undetectable in the RNA-Seq data surveyed. In this regard, the absence of *EgTIP3.2* expression was particularly intriguing, especially because its transcripts were readily detectable in leaves by RT-qPCR (**Figure 4**). Overall, these results reveal a distinctive spatial distribution of the *EgTIP* genes, indicating that certain isoforms may act in a relatively organ-specific manner.

### *EgTIP* Promoter Analysis Reveals the Presence of Stress-Related Regulatory Elements

Inspection of the 5′ upstream regions (1200 bp) of the *EgTIP* genes revealed the presence of several putative *cis*-regulatory elements mainly implicated in phytohormone and abiotic stress responses (**Table 2**). In this context, all *EgTIP* promoter regions, with the exception of *EgTIP1.2*, contained more than one ABA-responsive element (ABRE), involved in hormone responsiveness and in ABA-mediated abiotic stress signaling (Yamaguchi-Shinozaki and Shinozaki, 2005). The presence of ABRE in the promoters investigated suggests a possible role of ABA in the control of *EgTIP* expression as already reported for *HvTIP1.2* and *HvTIP3.1* in barley (Lee et al., 2015). Another well-represented regulatory element found in all the promoters investigated, except *EgTIP2.3*, was the TGACG-motif implicated in plant responses to methyl jasmonate (MeJA), a well-known primary signal in plant defense responses. Most *EgTIP* promoters (9 out of 11) also contained a MBS (MYB-binding site) motif generally recognized by MYB transcription factors implicated in drought and salt inducible gene expression. The circadian and SP1 elements known to play a role in circadian control and in light responses, respectively, were also detected in the majority of the *EgTIP* promoters. The circadian element is similarly over-represented in the promoter regions of aquaporin genes from sorghum (Reddy et al., 2015). Additional stress-related regulatory elements found in a small number of *EgTIP* promoters include TC-rich motifs, LTR, HSE and C-repeat/DRE (**Table 2**). Overall, these findings indicate that the *EgTIP* genes are regulated by phytohormones such as ABA and MeJA and differentially responsive to a set of abiotic stimuli. This prediction is supported by a previous study showing the modulation of *EgTIP2.2* promoter activity by osmotic stress and ABA (Rodrigues et al., 2013). However, the functionality of such predicted elements requires further validation.

**Table 2:** Main *cis*-regulatory elements found in the *EgTIP* promoters.

*EgTIP* genes	Element	Sequence	Function
1.1, 1.3, 1.4, 2.1, 2.2, 2.3, 3.1, 3.2, 4.1, 5.1	ABRE	ACGTG	*Cis*-acting element involved in the abscisic acid responsiveness
1.1, 1.2, 1.4, 2.1, 2.2, 2.3, 4.1	TC-RICH REPEATS	ATTTTCTTCA	*Cis*-acting element involved in defense and stress responsiveness
1.1, 1.2, 1.3, 1.4, 2.2, 4.1, 5.1	O2-SITE	GATGATGTGG	Element involved in metabolism regulation
1.1, 1.2, 1.3, 1.4, 2.1, 5.1	LTR	CCGAAA	Element involved in low-temperature responsiveness
1.1, 1.2, 2.1, 4.1	P-BOX	CCTTTTG	Gibberellin-responsive element
1.2, 1.4, 1.3, 3.2, 5.1	ATCT-motif	AATCTGATCG	Part of a conserved DNA module involved in light responsiveness
1.1, 1.2, 1.3, 1.4, 2.1, 2.2, 3.2, 4.1	CIRCADIAN	CAANNNNATC	Element involved in circadian control
1.1, 3.2, 5.1	HSE	AGAAAATTCG	Element involved in heat stress responsiveness
1.1, 1.2, 1.3, 1.4, 2.1, 2.3, 3.1, 4.1, 5.1	MBS	CAACTG	MYB binding site involved in drought-induction
1.1, 2.1, 2.2, 3.2	TCA-ELEMENT	CCATCTTTTT	Element involved in salicylic acid (SA) responsiveness
4.1, 5.1	SARE	TTCGACCTCCTT	Element involved in SA responsiveness
1.4, 2.1, 2.2, 2.3	TGA-ELEMENT	AACGAC	Auxin-responsive element
1.1, 1.4, 2.1, 3.2	ACE	AAAACGTTTA	Element involved in light responsiveness
1.4	C-repeat/DRE	TGGCCGAC	Element involved in cold and dehydration-responsiveness
1.2, 4.1, 5.1	Box-W1	TTGACC	Fungal elicitor responsive element
1.1, 1.2, 1.3, 1.4, 2.1, 2.2, 3.1, 3.2, 4.1, 5.1	TGACG-motif	TGACG	Element involved in the MeJA-responsiveness
1.1, 1.2, 1.3, 1.4, 2.1, 2.2, 3.1, 3.2, 4.1, 5.1	SP1	CC(G/A)CCC	Light responsive element


### *EgTIP* Expression Profiles Under PEG-Imposed Osmotic Stress

**FIGURE 6:**
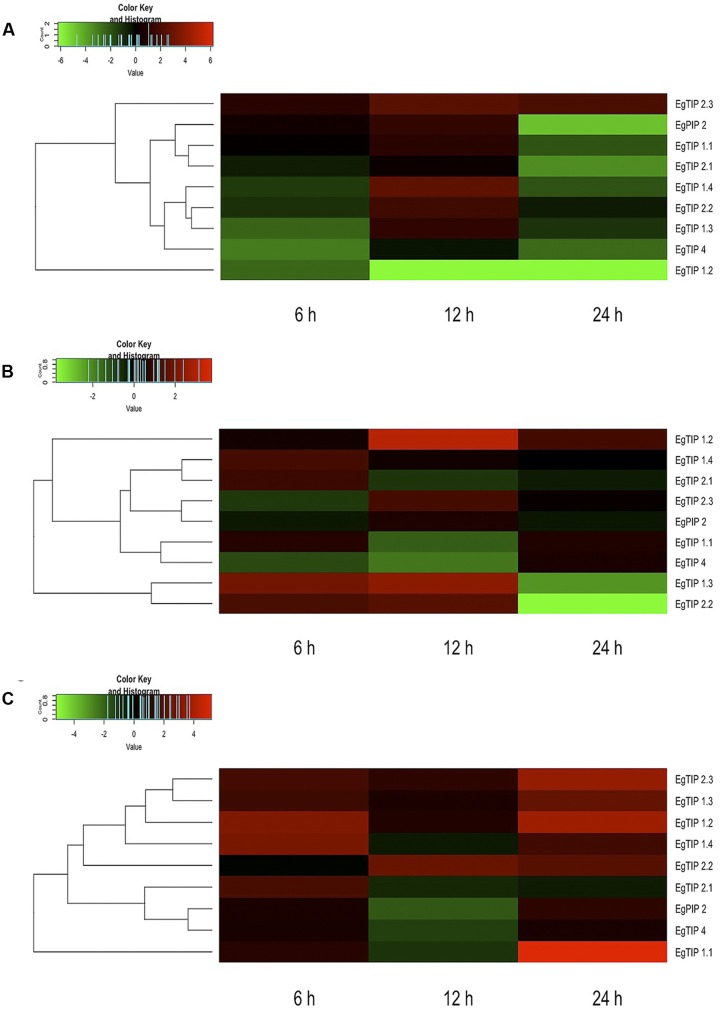
**Expression patterns of *EgTIP* genes under osmotic stress.** **(A)** Roots; **(B)** Stems; and **(C)** Leaves. The normalized fold change (log_2_ transformed) of *EgTIP* expression at each time point after PEG treatment was calculated relative to that of the untreated control at the same time point. The color scale is the same as in **Figure 4**. Genes were clustered according to their expression profiles. All samples were analyzed in triplicate.

Taking into account the aforementioned promoter analysis, we decided to evaluate the stress-responsive expression of each *EgTIP* gene. For this, total RNA extracted from roots, stems and leaves of Eucalyptus plantlets treated with PEG 8000 for 6, 12, and 24 h was employed. Relative to the untreated control, the majority of the investigated *EgTIPs*, with the obvious exception of the fruit-enriched *EgTIP3.1* and *EgTIP3.2* genes (not shown), showed increased expression upon PEG-induced osmotic stress at one or more time points (**Figure 6**). The timing and magnitude of the observed changes in gene expression were quite diverse among the investigated organs. In roots, most *EgTIPs* showed moderate fold-changes and reached peak expression levels mainly at 12 h of stress imposition (**Figure 6A**). In contrast, the timing of maximal expression in the stems varied between the investigated genes and was most evident at 6 and 12 h of PEG treatment (**Figure 6B**). *EgTIP1.2* showed maximum stress-induced fold-change in this organ at 12 h of PEG treatment. Compared to roots and stems, *EgTIP* expression was increased in leaves. In this organ, significant fold-changes were observed at 6 h, and more substantially, at 24 h of stress imposition (**Figure 6C**). At this time point, *EgTIP1.1*, *EgTIP1.2*, *EgTIP1.3*, and *EgTIP2.3* appeared as the most responsive genes. Interestingly, despite being barely detectable in leaves and stems, *EgTIP2.3* was moderately and highly induced by osmotic stress in the investigated organs. In addition, the expression of *EgPIP2*, which was used as an inducible control (Rodrigues et al., 2013), was monitored in parallel and shown to be induced by PEG in all organs examined.

## Discussion

Although described throughout all kingdoms, aquaporins show a particularly high diversity and abundance in plants. Among the plant aquaporins, the members of the TIP subfamily are implicated in the control of tonoplast permeability to water and other solutes. Despite the accumulated knowledge in model species, much less is known about the organization of the TIP subfamily in woody species, especially in Eucalyptus, an economically important and high water-demanding forest tree.

In this study, we show that 11 members, which have been assigned to the five classical TIP groups of higher plants, compose the TIP subfamily of Eucalyptus. These data expand the results of Rodrigues et al. (2013), revealing the presence of six additional TIP genes in *E. grandis*. When compared to other woody species, the Eucalyptus TIP subfamily is almost the same size as those described in *V. vinifera* (10 genes) and *C. sinensis* (11 genes), but possess less members than *Populus* and rubber tree (17 genes each). The number of TIP genes in the Eucalyptus genome is also well conserved among other well-studied model species such as *Arabidopsis* (10 genes) and rice (10 genes). Thus, despite the occurrence of a whole-genome duplication (WGD) event and the reported large number of tandem duplications (Myburg et al., 2014), no major expansion of the eucalyptus TIP subfamily relatively to other woody and non-woody species was observed. This is in clear contrast with *Populus*, in which the expansion of the MIP superfamily, including the acquisition of several TIP genes, has been attributed to gene retention after a recent WGD event (Gupta and Sankararamakrishnan, 2009).

Concerning their structure, the *EgTIP* genes showed a similar pattern of exon/intron organization as reported for other TIP genes from dicot species that normally harbor three exons separated by two introns. The only exception is the single intron-containing *EgTIP1.1* gene, which probably evolved from sequential loss of introns as previously suggested (Regon et al., 2014).

### *EgTIP* Expression Patterns and Possible Functional Diversification

Based on our results, distinct organ/tissue expression profiles were observed for the *EgTIP* genes identified. Among them, *EgTIP1.1* was the most widely expressed, indicating that it might play a more generalized role throughout the plant. This is in line with previous reports showing that TIP1.1 is widely distributed and the most abundantly expressed TIP isoform (Alexandersson et al., 2005; Knipfer et al., 2011; Regon et al., 2014). Conversely, the other *EgTIP* genes showed enriched-organ/tissue expression, a feature that was particularly evident in wood-related and reproductive organs/tissues. The only exception was *EgTIP5.1*, which appears to be nonfunctional since no transcripts or expression sequence tag evidences could be found for this gene. Interestingly, none of the identified *EgTIP* genes was found to be exclusively expressed in a specific organ/tissue.

Despite some partial overlaps, the observed divergences in their spatial expression patterns indicate that *EgTIPs* may have undergone functional diversification. This fact also suggests that certain isoforms might have acquired specific organ/tissue roles. Evidence from poplar trees, however, indicate that, in spite of their elevated tissue-specificity, a high level of functional redundancy exists amongst *PtTIP* members (Cohen et al., 2013). In this respect, the existence of functional redundancy between TIP genes was postulated in previous studies employing insertional *Arabidopsis* mutants (Schüssler et al., 2008). In general, current evidences indicate that TIP isoforms act redundantly to regulate water and solute transport. In contrast with this, Gattolin et al. (2009) demonstrated the existence of tissue- and cell-specific patterns of expression of some *AtTIP* genes in *Arabidopsis* roots. Similarly, a specific and developmentally regulated leaf expression pattern of some *HvTIP* subfamily members was observed in barley (Besse et al., 2011). These results highlight the existence of possible specialization of TIP isoforms, but further investigations are required to uncover possible functional specificity of the identified *EgTIP* genes.

In trees, the modulation of *TIP* expression in response to abiotic stress has been documented (Cohen et al., 2013; Rodrigues et al., 2013; Martins et al., 2015; Regon et al., 2014), and the major evidences point to a role for these proteins in stress adaptation. It is noteworthy that most of the *EgTIP* genes analyzed here displayed osmotic stress-responsive transcriptional profiles in three different organs. Although the timing and extent of their expression varied, these results indicate that the Eucalyptus responses to osmotic stress require the coordinated transcriptional regulation of most *EgTIPs*. The existence of *cis*-regulatory elements related to hormone and abiotic stress response in the *EgTIP* promoter regions lend further support to this idea. Consistently, we have previously shown the induction and repression of the *EgTIP2.2* promoter activity by osmotic stress and ABA, respectively (Rodrigues et al., 2013). However, despite these reported evidences of transcriptional regulation, the possible contribution of the different EgTIP isoforms in the control of water flow under stress conditions requires further investigation.

### EgTIP Substrate Selectivity

The selectivity filter of aquaporins comprises four residues located at the non-cytosolic portion of the pore. These residues are implicated in water translocation and in the selection of other substrate molecules such as glycerol, ammonia, boric acid, CO_2_, H_2_O_2_, and urea (reviewed in Maurel et al., 2015). Previous data have indicated that TIP subfamily members show the highest level of sequence divergence in the ar/R region among known plant aquaporins (Wallace and Roberts, 2004; Azad et al., 2012). Despite this, some residues are well conserved, particularly in the LE1 position, in which an A or G is present, and in the H_2_ position, in which an H residue has been associated with water specificity (Sui et al., 2001; Kirscht et al., 2016). Remarkably, this conserved H residue is found in most identified EgTIPs, with the exception of EgTIP3.2 and EgTIP5.1.

Concerning LE2, an R residue known to contribute to the mechanism of proton exclusion is usually present at this position (Wu et al., 2009; Kirscht et al., 2016). In TIPs, however, this residue can be replaced by polar residues such as C or nonpolar residues such as V, a feature that is observed in some EgTIPs. Intriguingly, a nonpolar L residue in found in this position in EgTIP3.1. According to Azad et al. (2012), the presence of a hydrophobic residue in this position in TIP1 homologs renders the proteins multifunctional aqua-glyceroporins. Thus, changes in aa charge and side chains lead to modifications in pore size and hydrophobicity that have direct consequences in TIP transport selectivity.

A still unresolved question concerns EgTIP2.3. Due to its clustering with known TIP5 isoforms, EgTIP2.3 could be also assigned as a member of group 5. However, EgTIP2.3 lack the tetrad NVGC commonly found in the ar/R filter of most TIP5 isoforms. Instead, it harbors a selectivity filter that resembles the one present in TIP2 isoforms, an attribute that justifies its nomination as a TIP2 in Phytozome. The closest homologue of EgTIP2.3, VvTIP5.2, also harbors a canonic TIP2-like ar/R filter (HIGR). Intriguingly, such an ambiguous classification was also observed for certain NOD26-like intrinsic proteins from different plant species (Zou et al., 2015b). Overall, our results suggest that both EgTIP2.3 and VvTIP5.2 represent unusual isoforms sharing phylogenetic relationships with TIP5s but transport selectivity of TIP2s.

Several studies have demonstrated that additional residues may contribute to TIP preference for the transport of other substrates than water (Froger et al., 1998; Hove and Bhave, 2011). A recent study that elucidated the crystal structure of AtTIP2.1 proposed the expansion of the selectivity filter with the inclusion of a flexible H residue in loop C (Kirscht et al., 2016), that has been implicated in ammonia transport. In this respect, EgTIP2.1, EgTIP2.2, and EgTIP4.1 have the same H residue in loop C as AtTIP2.1. Comparatively, several TIP2 orthologs from other plant species also have an AtTIP2.1-like extended selectivity filter (Zou et al., 2015a,b, 2016), thus suggesting that these proteins may be able to transport ammonia. Moreover, in view of the predictions for non-aqua transport profiles proposed by Azad et al. (2016), both EgTIP2 and EgTIP4.1 as well as all EgTIP1 fulfill the requirements to act as H_2_O_2_ transporters.

Previous reports have also included the analysis of nine specificity-determining positions (SDPs) to predict non-aqua substrates (Hove and Bhave, 2011) as well as the Froger’s positions (P1-P5) to discriminate TIP-mediated glycerol transport (Froger et al., 1998). All these positions were evaluated in the EgTIPs investigated in order to address possible transport specificity. Although the Froger’s positions remained highly conserved in EgTIPs compared to TIPs found in other plant species (**Table 1**), they do not fit the requirements for glycerol transport. Noteworthy, as reported earlier for TIPs from *Ricinus communis* and *Jatropha curcas* (Zou et al., 2015b, 2016), the SDP analysis indicated the presence of residues typically associated with urea transport in all EgTIPs (**Supplementary Table S4**). In particular, EgTIP1.1, EgTIP4.1, and EgTIP5.1 have classical urea-type SDPs (**Supplementary Table S4**). Overall, these results are valuable for future studies on the structure–function relationships of the identified EgTIPs.

## Conclusion

In this work, an in depth analysis of the Eucalyptus TIP subfamily was performed using different molecular approaches. Eleven genes were identified based upon sequence similarity and phylogenetic relationships with known TIPs from other plant species. Among them, only *EgTIP5.1* was not expressed and seems not to be functional. The structural and functional properties of the EgTIP isoforms were further investigated using bioinformatics tools, and their capacities to transport water and other substrates were predicted and discussed. Interestingly, distinctive spatial expression patterns and inducible expression under osmotic stress were observed. Although to be taken with caution, a possible involvement of the *EgTIP* products in abiotic stress responses was envisaged. The genes identified in this study represent an important resource for further functional analyses and use in biotechnological programs aiming Eucalyptus genetic improvement.

## Author Contributions

Conceived and designed the experiments: MR, AT, and IM; performed the experiments: MR and AT; analyzed the data: JB and IM; wrote the paper: MR, AT, and IM.

## Conflict of Interest Statement

The authors declare that the research was conducted in the absence of any commercial or financial relationships that could be construed as a potential conflict of interest.

## Footnotes

1 http://www.phytozome.net/

2 http://gsds.cbi.pku.edu.cn/

3 http://chibba.agtec.uga.edu/duplication/

4 http://www.csbio.sjtu.edu.cn/bioinf/plant-multi/\#

5 http://www.clustal.org/

6 http://www.megasoftware.net/

7 www.phytozome.net

## References

[B1] Afzal Z. Howton T. C. Sun Y. Mukhtar M. S. (2016). The roles of aquaporins in plant stress responses. *J. Dev. Biol.* 4 9 10.3390/jdb4010009 PMC5831814 29615577

[B2] Alexandersson E. Fraysse L. Sjövall-Larsen S. Gustavsson S. Fellert M. Karlsson M. (2005). Whole gene family expression and drought stress regulation of aquaporins. *Plant Mol. Biol.* 59 469–484. 10.1007/s11103-005-0352-1 16235111

[B3] Ashkenazy H. Erez E. Martz E. Pupko T. Ben-Tal N. (2010). ConSurf 2010: calculating evolutionary conservation in sequence and structure of proteins and nucleic acids. *Nucleic Acids Res.* 38 W529–33. 10.1093/nar/gkq399 20478830 PMC2896094

[B4] Azad A. K. Ahmed J. Alum M. A. Hasan M. M. Ishikawa T. Sawa Y. (2016). Genome-wide characterization of major intrinsic proteins in four grass plants and their non-Aqua transport selectivity profiles with comparative perspective. *PLoS ONE* 11:e0157735 10.1371/journal.pone.0157735 PMC4915720 27327960

[B5] Azad A. K. Yoshikawa N. Ishikawa T. Sawa Y. Shibata H. (2012). Substitution of a single amino acid residue in the aromatic/arginine selectivity filter alters the transport profiles of tonoplast aquaporin homologs. *Biochim. Biophys. Acta* 1818 1–11. 10.1016/j.bbamem.2011.09.014 21963407

[B6] Besse M. Knipfer T. Miller A. J. Verdeil J. L. Jahn T. P. Fricke W. (2011). Developmental pattern of aquaporin expression in barley (*Hordeum vulgare* L.) leaves. *J. Exp. Bot.* 62 4127–4142. 10.1093/jxb/err175 21737414 PMC3153690

[B7] Boursiac Y. Chen S. Luu D. T. Sorieul M. Van den Dries N. Maurel C. (2005). Early effects of salinity on water transport in *Arabidopsis* roots. molecular cellular features of aquaporin expression. *Plant Physiol.* 139 k790–805. 10.1104/pp.105.065029 PMC1255996 16183846

[B8] Chou K.-C. Shen H.-B. (2010). Plant-mPLoc: a top-down strategy to augment the power for predicting plant protein subcellular localization. *PLoS ONE* 5:e11335 10.1371/journal.pone.0011335 PMC2893129 20596258

[B9] Cohen D. Bogeat-Triboulot M. B. Vialet-Chabrand S. Merret R. Courty P. E. Moretti S. (2013). Developmental and environmental regulation of Aquaporin gene expression across *Populus* species: divergence or redundancy? *PLoS ONE* 8:e55506 10.1371/journal.pone.0055506 PMC3564762 23393587

[B10] de Oliveira L. A. Breton M. C. Bastolla F. M. Camargo Sda S. Margis R. Frazzon J. (2012). Reference genes for the normalization of gene expression in eucalyptus species. *Plant Cell Physiol.* 53 405–422. 10.1093/pcp/pcr187 22197885 PMC7107212

[B11] Finn R. D. Coggill P. Eberhardt R. Y. Eddy S. R. Mistry J. Mitchell A. L. (2016). The Pfam protein families database: towards a more sustainable future. *Nucleic Acids Res.* 44 D279–D285. 10.1093/nar/gkv1344 26673716 PMC4702930

[B12] Froger A. C. Tallur B. C. Thomas D. C. Delamarche C. (1998). Prediction of functional residues in water channels and related proteins. *Protein Sci.* 7 1458–1468. 10.1002/pro.5560070623 9655351 PMC2144022

[B13] Gattolin S. Sorieul M. Hunter P. R. Khonsari R. H. Frigerio L. (2009). In vivo imaging of the tonoplast intrinsic protein family in *Arabidopsis* roots. *BMC Plant Biol.* 9:133 10.1186/1471-2229-9-133 PMC2784467 19922653

[B14] Goicoechea M. Lacombe E. Legay S. Mihaljevic S. Rech P. Jauneau A. (2005). EgMYB2, a new transcription activator from *Eucalyptus* xylem, regulates secondary cell wall formation and lignin biosynthesis. *Plant J.* 43 553–567. 10.1111/j.1365-313X.2005.02480.x 16098109

[B15] Gupta A. B. Sankararamakrishnan R. (2009). Genome-wide analysis of major intrinsic proteins in the tree plant *Populus trichocarpa*: characterization of XIP subfamily of aquaporins from evolutionary perspective. *BMC Plant Biol.* 9:134 10.1186/1471-2229-9-134 PMC2789079 19930558

[B16] Hove R. M. Bhave M. (2011). Plant aquaporins with non-aqua functions: deciphering the signature sequences. *Plant Mol. Biol.* 75 413–430. 10.1007/s11103-011-9737-5 21308399

[B17] Johanson U. Karlsson M. Johansson I. Gustavsson S. Sjövall S. Fraysse L. (2001). The complete set of genes encoding major intrinsic proteins in *Arabidopsis* provides a framework for a new nomenclature for major intrinsic proteins in plants. *Plant Physiol.* 126 1358–1369. 10.1104/pp.126.4.1358 11500536 PMC117137

[B18] Kelley L. A. Mezulis S. Yates C. M. Wass M. N. Sternberg M. J. E. (2015). The Phyre2 web portal for protein modeling, prediction and analysis. *Nat. Protoc.* 10 845–858. 10.1038/nprot.2015.053 25950237 PMC5298202

[B19] Khan K. Agarwal P. Shanware A. Sane V. A. (2015). Heterologous expression of two Jatropha aquaporins imparts drought and salt tolerance and improves seed viability in transgenic *Arabidopsis thaliana*. *PLoS ONE* 10:e0128866 10.1371/journal.pone.0128866 PMC4466373 26067295

[B20] Kirscht A. Kaptan S. S. Bienert G. P. Chaumont F. Nissen P. de Groot B. L. (2016). Crystal structure of an ammonia-permeable aquaporin. *PLoS Biol.* 14:e1002411 10.1371/journal.pbio.1002411 PMC4814140 27028365

[B21] Knipfer T. Besse M. Verdeil J. L. Fricke W. (2011). Aquaporin-facilitated water uptake in barley (*Hordeum vulgare* L.) roots. *J. Exp. Bot.* 62 4115–4126. 10.1093/jxb/err075 21441404 PMC3153672

[B22] Landau M. Mayrose I. Rosenberg Y. Glaser F. Martz E. Pupko T. (2005). ConSurf 2005: the projection of evolutionary conservation scores of residues on protein structures. *Nucleic Acids Res.* 33 W299–W302. 10.1093/nar/gki370 15980475 PMC1160131

[B23] Lee S. E. Yim H. K. Lim M. N. Yoon I. S. Kim J. H. Hwang Y. S. (2015). Abscisic acid prevents the coalescence of protein storage vacuoles by upregulating expression of a tonoplast intrinsic protein gene in barley aleurone. *J. Exp. Bot.* 66 1191–1203. 10.1093/jxb/eru467 25477530 PMC4438444

[B24] Li J. Cai W. (2015). A ginseng PgTIP1 gene whose protein biological activity related to Ser(128) residue confers faster growth and enhanced salt stress tolerance in *Arabidopsis*. *Plant Sci.* 234 74–85. 10.1016/j.plantsci.2015.02.001 25804811

[B25] Lin W. Peng Y. Li G. Arora R. Tang Z. Su W. (2007). Isolation and functional characterization of PgTIP1, a hormone-autotrophic cells-specific tonoplast aquaporin in ginseng. *J. Exp. Bot.* 58 947–956. 10.1093/jxb/erl255 17237160

[B26] Liu L. H. Ludewig U. Gassert B. Frommer W. B. von Wirén N. (2003). Urea transport by nitrogen-regulated tonoplast intrinsic proteins in *Arabidopsis*. *Plant Physiol.* 133 1220–1228. 10.1104/pp.103.027409 14576283 PMC281617

[B27] Livak K. Schmittgen T. D. (2001). Analysis of relative gene expression data using real time quantitative PCR and the 2^-ΔΔCt^ method. *Methods* 25 402–408. 10.1006/meth.2001.1262 11846609

[B28] Loqué D. Ludewig U. Yuan L. von Wirén N. (2005). Tonoplast intrinsic proteins AtTIP2;1 and AtTIP2;3 facilitate NH3 transport into the vacuole. *Plant Physiol.* 137 671–680. 10.1104/pp.104.051268 15665250 PMC1065367

[B29] Lovell S. C. Davis I. W. Arendall W. B. III de Bakker P. I. W. Word J. M. Prisant M. G. (2003). Structure validation by Calpha geometry: phi, psi and Cbeta deviation. *Proteins* 50 437–450. 10.1002/prot.10286 12557186

[B30] Maeshima M. (2001). TONOPLAST TRANSPORTERS: organization and function. *Annu. Rev. Plant Physiol. Plant Mol. Biol.* 52 469–497. 10.1146/annurev.arplant.52.1.469 11337406

[B31] Martins C. de P. Pedrosa A. M. Du D. Gonçalves L. P. Yu Q. (2015). Genome-wide characterization and expression analysis of major intrinsic proteins during abiotic and biotic stresses in sweet orange (Citrus sinensis L. Osb.). *PLoS ONE* 10:e0138786 10.1371/journal.pone.0138786 PMC4580632 26397813

[B32] Marti-Renom M. A. Stuart A. Fiser A. Sánchez R. Melo F. Sali A. (2000). Comparative protein structure modeling of genes and genomes. *Annu. Rev. Biophys. Biomol. Struct.* 29 291–325. 10.1146/annurev.biophys.29.1.291 10940251

[B33] Maurel C. Boursiac Y. Luu D. T. Santoni V. Shahzad Z. Verdoucq L. (2015). Aquaporins in plants. *Physiol. Rev.* 95 1321–1358. 10.1152/physrev.00008.2015 26336033

[B34] Mizrachi E. Hefer C. A. Ranik M. Joubert F. Myburg A. A. (2010). De novo assembled expressed gene catalog of a fast-growing Eucalyptus tree produced by Illumina mRNA-Seq. *BMC Genomics* 11:681 10.1186/1471-2164-11-681 PMC3053591 21122097

[B35] Myburg A. A. Grattapaglia D. Tuskan G. A. Hellsten U. Hayes R. D. Grimwood J. (2014). The genome of *Eucalyptus grandis*. *Nature* 510 356–362. 10.1038/nature13308 24919147

[B36] Peng Y. Lin W. Cai W. Arora R. (2007). Overexpression of a *Panax* ginseng tonoplast aquaporin alters salt tolerance, drought tolerance and cold acclimation ability in transgenic *Arabidopsis* plants. *Planta* 226 729–740. 10.1007/s00425-007-0520-4 17443343

[B37] Ramakers C. Ruijtera J. M. Ronald H. Lekanne D. Moormana A. F. M. (2003). Assumption-free analysis of quantitative real-time polymerase chain reaction (PCR) data. *Neurosci. Lett.* 339 62–66. 10.1016/S0304-3940(02)01423-4 12618301

[B38] Reddy P. S. Rao T. S. R. B. Sharma K. K. Vadez V. (2015). Genome-wide identification and characterization of the aquaporin gene family in *Sorghum bicolor* (L.). *Plant Gene.* 1 18–28. 10.1016/j.plgene.2014.12.002

[B39] Regon P. Panda P. Kshetrimayum E. Panda S. K. (2014). Genome-wide comparative analysis of tonoplast intrinsic protein (TIP) genes in plants. *Funct. Integr. Genomics* 14 617–629. 10.1007/s10142-014-0389-9 25095751

[B40] Reinhardt H. Hachez C. Bienert M. D. Beebo A. Swarup K. Voß U. (2016). Tonoplast aquaporins facilitate lateral root emergence. *Plant Physiol.* 170 1640–1654. 10.1104/pp.15.01635 26802038 PMC4775129

[B41] Rodrigues M. I. Bravo J. P. Sassaki F. T. Severino F. E. Maia I. G. (2013). The tonoplast intrinsic aquaporin (TIP) subfamily of *Eucalyptus grandis*: characterization of EgTIP2, a root-specific and osmotic stress-responsive gene. *Plant Sci.* 213 106–113. 10.1016/j.plantsci.2013.09.005 24157213

[B42] Sade N. Vinocur B. J. Diber A. Shatil A. Ronen G. Nissan H. (2009). Improving plant stress tolerance and yield production: is the tonoplast aquaporin SlTIP2;2 a key to isohydric to anisohydric conversion? *New Phytol.* 181 651–661. 10.1111/j.1469-8137.2008.02689.x 19054338

[B43] Sali A. Blundell T. L. (1993). Comparative protein modelling by satisfaction of spatial restraints. *J. Mol. Biol.* 234 779–815. 10.1006/jmbi.1993.1626 8254673

[B44] Schrödinger L. C. C. (2011). *The PyMol Molecular Graphics System. Version 1.3.* Available at: http://www.pymol.org/

[B45] Schüssler M. D. Alexandersson E. Bienert G. P. Kichey T. Laursen K. H. Johanson U. (2008). The effects of the loss of TIP1;1 and TIP1;2 aquaporins in *Arabidopsis thaliana*. *Plant J.* 56 756–767. 10.1111/j.1365-313X.2008.03632.x 18643996

[B46] Shelden M. C. Howitt S. M. Kaiser B. N. Tyerman S. D. (2009). Identification and functional characterisation of aquaporins in the grapevine, *Vitis vinifera*. *Funct. Plant Biol.* 36 1065–1078. 10.1071/FP09117 32688718

[B47] Sui H. Han B. G. Lee J. K. Walian P. Jap B. K. (2001). Structural basis of water-specific transport through the AQP1 water channel. *Nature* 414 872–878. 10.1038/414872a 11780053

[B48] Wallace I. S. Roberts D. M. (2004). Homology modeling of representative subfamilies of *Arabidopsis* major intrinsic proteins. Classification based on the aromatic/arginine selectivity filter. *Plant Physiol.* 135 1059–1068. 10.1104/pp.103.033415 15181215 PMC514140

[B49] Wang X. Li Y. Ji W. Bai X. Cai H. Zhu D. (2011). A novel *Glycine soja* tonoplast intrinsic protein gene responds to abiotic stress and depresses salt and dehydration tolerance in transgenic *Arabidopsis thaliana*. *J. Plant Physiol.* 168 1241–1248. 10.1016/j.jplph.2011.01.016 21397356

[B50] Wu B. Steinbronn C. Alsterfjord M. Zeuthen T. Beitz E. (2009). Concerted action of two cation filters in the aquaporin water channel. *EMBO J.* 28 2188–2194. 10.1038/emboj.2009.182 19574955 PMC2726698

[B51] Yamaguchi-Shinozaki K. Shinozaki K. (2005). Organization of cis-acting regulatory elements in osmotic- and cold-stress-responsive promoters. *Trends Plant Sci.* 10 88–94. 10.1016/j.tplants.2004.12.012 15708346

[B52] Yu H. Soler M. Mila I. San Clemente H. Savelli B. Dunand C. (2014). Genome-wide characterization and expression profiling of the AUXIN RESPONSE FACTOR (ARF) gene family in *Eucalyptus grandis*. *PLoS ONE* 9:e108906 10.1371/journal.pone.0108906 PMC4182523 25269088

[B53] Zou Z. Gong J. An F. Xie G. Wang J. Mo Y. (2015a). Genome-wide identification of rubber tree (*Hevea brasiliensis* Muell. Arg.) aquaporin genes and their response to ethephon stimulation in the laticifer, a rubber-producing tissue. *BMC Genomics* 16:1001 10.1186/s12864-015-2152-6 PMC4658816 26606923

[B54] Zou Z. Gong J. Huang Q. Mo Y. Yang L. Xie G. (2015b). Gene structures, evolution, classification and expression profiles of the aquaporin gene family in castor bean (*Ricinus communis* L.). *PLoS ONE* 10:e0141022 10.1371/journal.pone.0141022 PMC4625025 26509832

[B55] Zou Z. Yang L. Gong J. Mo Y. Wang J. Cao J. (2016). Genome-wide identification of *Jatropha curcas* aquaporin genes and the comparative analysis provides insights into the gene family expansion and evolution in *Hevea brasiliensis*. *Front. Plant Sci.* 7:395 10.3389/fpls.2016.00395 PMC4814485 27066041

