# Supplementary material for: The Eucalyptus Tonoplast Intrinsic Protein (TIP) Gene Subfamily: Genomic Organization, Structural Features, and Expression Profiles
Source: Front Plant Sci. 2016 Nov 30;7:1810. doi: 10.3389/fpls.2016.01810 (PMC5127802; doi:10.3389/fpls.2016.01810)
Supplement: Supplementary file 1 [file Table_1.DOC]

Table S1. List of primers used in gene expression analysis by RT-qPCR.

| **Primer Name** | **Sequence** | **Tm (^o^C)** | **GC Content (%)** |
| --- | --- | --- | --- |
| **RT ACT F** | GTC TTT CCC AGT ATT GTT GGT | 56 | 43 |
| **RT ACT R** | AAC ATA GGC ATC CTT CTG AC | 54 | 45 |
| **RT TIP1.1 F** | GGTCCACCGTCGCTTGCTT | 65.9 | 63.16 |
| **RT TIP1.1 R** | GATGGCCGTGGCGTACACA | 66.45 | 63.16 |
| **RT TIP1.2 F** | CCATGCATTCGGGCTGTT | 63.03 | 55.56 |
| **RT TIP1.2 R** | AGATGCCCCTCAACAAGGTT | 60.88 | 50 |
| **RT TIP1.3 F** | TCAGTTGCTTGGTTCGGTAGTG | 62.49 | 50 |
| **RT TIP1.3 R** | AAAGACCAGTGCATTCCACA | 59.14 | 45 |
| **RT TIP1.4 F** | CAGGCCGATACCCTCAAA | 59,6 | 55.56 |
| **RT TIP1.4 R** | AACAGCGCAAAGGCGTGA | 64.09 | 55.56 |
| **RT TIP2.1 F** | TGTTGCAGTTGCAGTGGGT | 61.37 | 52.63 |
| **RT TIP2.1 R** | AACTATGGCGCCAACAAGC | 61.18 | 52.63 |
| **RT TIP2.2 F** | TGG CTT TGG TGA CTC CTT C | 59.00 | 53 |
| **RT TIP2.2 R** | CGG CGA ACA CGA AGA GG | 62.00 | 65 |
| **RT TIP3.1 F** | TTGGGTGCCATACTAGCCTCA | 62.81 | 52.38 |
| **RT TIP3.1 R** | CTATTTCTAGGAGCAGCCCGT | 59.88 | 52.38 |
| **RT TIP3.2 F** | TTGAGGGAGCTCCTCTTGGA | 62.36 | 55 |
| **RT TIP3.2 R** | AGGATGTTCGCCCCTAGGAT | 62.07 | 55 |
| **RT TIP4.1 F** | GTTGTCCGATCGGTCCTGTAT | 61,15 | 52.38 |
| **RT TIP4.1 R** | CAAGGGTATGTACTGGTGTAGCC | 59,83 | 52.17 |
| **RT TIP2.3 F** | CATGTCAACCCAGCAGTGAC | 60.16 | 55 |
| **RT TIP2.3 R** | GCATCAAGAGGACACAAGCA | 60 | 50 |
