# Supplementary material for: The Eucalyptus Tonoplast Intrinsic Protein (TIP) Gene Subfamily: Genomic Organization, Structural Features, and Expression Profiles
Source: Front Plant Sci. 2016 Nov 30;7:1810. doi: 10.3389/fpls.2016.01810 (PMC5127802; doi:10.3389/fpls.2016.01810)
Supplement: Supplementary file 2 [file Table_2.DOCX]

**Supplementary Table S2.** Synonymous (Ks) and non-synonymous (Ka) substitution rates of the identified EgTIP gene pairs.

| No | Gene 1 | Gene 2 | Ka | Ks | Ka/Ks |
| --- | --- | --- | --- | --- | --- |
| 1 | EgTIP1.3 | EgTIP1.4 | 0,06 | 1,37 | 0,0438 |
| 2 | EgTIP3.2 | EgTIP3.1 | 0,14 | 1,61 | 0,0870 |
| 3 | EgTIP4.1 | EgTIP2.1 | 0,39 | 1,62 | 0,2407 |
| 4 | EgTIP2.2 | EgTIP2.3 | 0,45 | 1,33 | 0,3383 |
