# Supplementary material for: The Eucalyptus Tonoplast Intrinsic Protein (TIP) Gene Subfamily: Genomic Organization, Structural Features, and Expression Profiles
Source: Front Plant Sci. 2016 Nov 30;7:1810. doi: 10.3389/fpls.2016.01810 (PMC5127802; doi:10.3389/fpls.2016.01810)
Supplement: Supplementary file 3 [file Table_3.DOCX]

**Supplementary Table S3.** Ramachandran plot results for the *E. grandis* TIP models

| **Protein** | **Favored region (%)** | **Allowed region (%)** | **Disallowed region (%)** |
| --- | --- | --- | --- |
| **EgTIP1.1** | 98.3 | 1.7 | 0 |
| **EgTIP2.1** | 97.9 | 2.1 | 0 |
| **EgTIP3.1** | 97.5 | 2.5 | 0 |
| **EgTIP4.1** | 97.4 | 2.6 | 0 |
| **EgTIP5.1** | 96.9 | 2.2 | 0.9 |
