# Supplementary material for: The Eucalyptus Tonoplast Intrinsic Protein (TIP) Gene Subfamily: Genomic Organization, Structural Features, and Expression Profiles
Source: Front Plant Sci. 2016 Nov 30;7:1810. doi: 10.3389/fpls.2016.01810 (PMC5127802; doi:10.3389/fpls.2016.01810)
Supplement: Supplementary file 4 [file Table_4.DOCX]

**Supplementary Table S4.** SPD residues identified in the investigated EgTIPs^*^.

|  | **SPD1** | **SPD2** | **SPD3** | **SPD4** | **SPD5** | **SPD6** | **SPD7** | **SPD8** | **SPD9** |
| --- | --- | --- | --- | --- | --- | --- | --- | --- | --- |
| **Typical Urea Transporter** | **H** | **P** | **F/I/L/T** | **A/C/F/L** | **L/M** | **A/G/P** | **G/S** | **G/S** | **N** |
| **EgTIP1.1** | H | P | F | F | L | A | G | S | N |
| **EgTIP1.2** | H | P | F | F | L | A | G | S | N |
| **EgTIP1.3** | H | P | F | F | L | A | G | S | N |
| **EgTIP1.4** | H | P | F | F | L | A | G | S | N |
| **EgTIP2.1** | H | P | F | A | L | P | G | S | N |
| **EgTIP2.2** | H | P | L | A | L | P | G | S | N |
| **EgTIP2.3** | H | P | M | V | L | P | G | F | N |
| **EgTIP3.1** | H | P | L | L | L | P | G | S | N |
| **EgTIP3.2** | H | P | F | L | L | P | G | S | N |
| **EgTIP4.1** | H | P | L | L | L | A | G | S | N |
| **EgTIP5.1** | H | P | F | A | L | P | G | S | N |
| **Typical H_2_O_2_ Transporter** | **A/S** | **A/G** | **L/V** | **A/F/L/T/V** | **I/L/V** | **H/I/L/Q** | **F/Y** | **A/V** | **P** |
| **EgTIP1.1** | S | A | L | A | I | H | Y | A | P |
| **EgTIP1.2** | S | A | L | A | I | H | Y | **L** | P |
| **EgTIP1.3** | S | A | L | **S** | I | H | Y | **L** | P |
| **EgTIP1.4** | S | A | L | A | I | H | Y | **L** | P |
| **EgTIP2.1** | A | A | L | V | I | **N** | Y | V | P |
| **EgTIP2.2** | S | A | L | V | I | **N** | Y | V | P |
| **EgTIP2.3** | S | A | L | L | V | H | H | V | P |
| **EgTIP3.1** | A | A | L | V | I | H | Y | **L** | P |
| **EgTIP3.2** | A | A | L | **I** | I | H | Y | **L** | P |
| **EgTIP4.1** | S | A | L | L | V | H | Y | V | P |
| **EgTIP5.1** | A | A | L | A | I | Q | Y | V | P |

*Residues of EgTIPs that do not correspond to the typical SPDs are shown in bold.
