# Supplementary figures and images for: The Eucalyptus Tonoplast Intrinsic Protein (TIP) Gene Subfamily: Genomic Organization, Structural Features, and Expression Profiles
Source: Front Plant Sci. 2016 Nov 30;7:1810. doi: 10.3389/fpls.2016.01810 (PMC5127802; doi:10.3389/fpls.2016.01810)

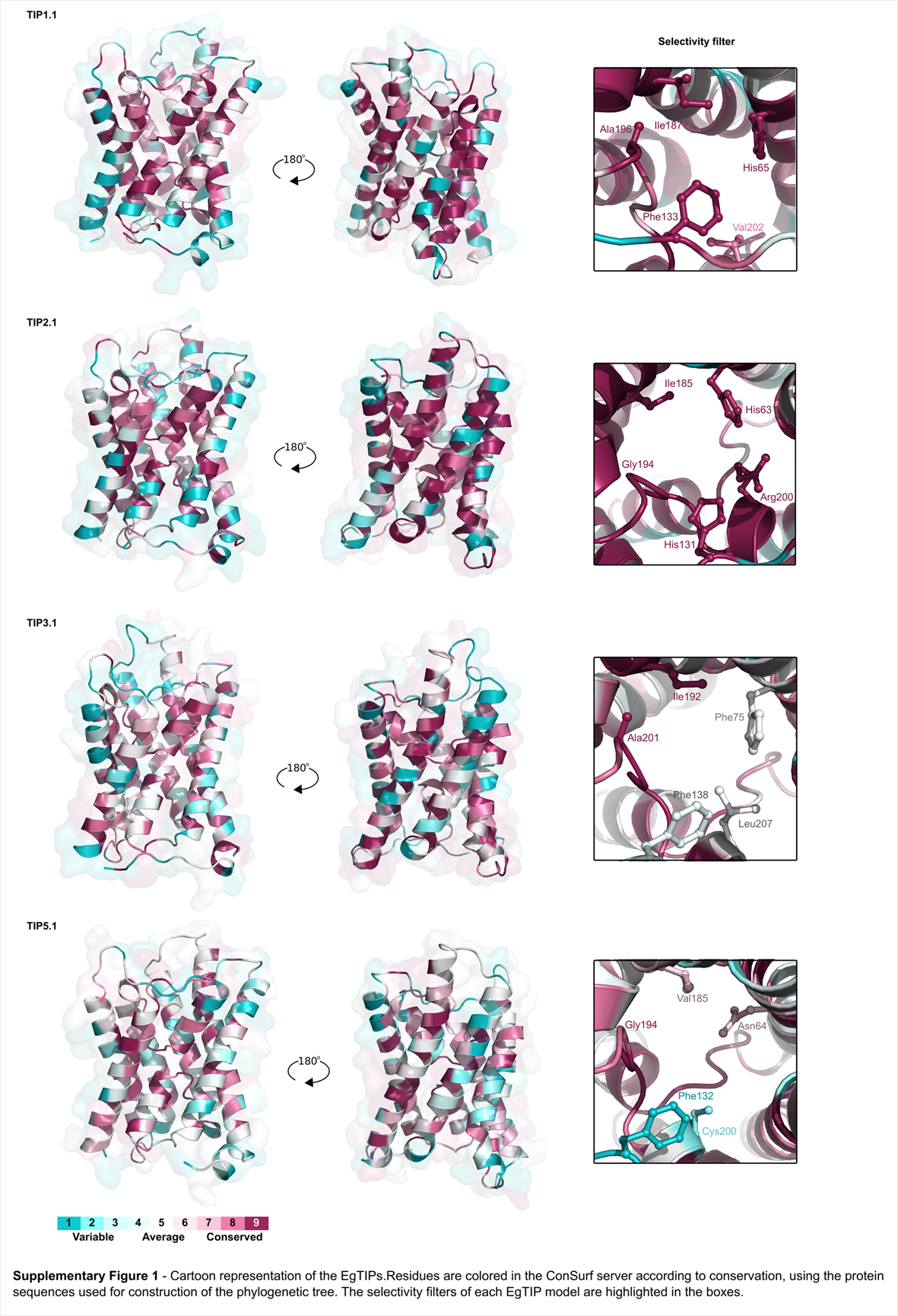

Supplement: Supplementary file 5 [file Image_1.TIF]
